# A method to benchmark the balance resilience of robots

**DOI:** 10.3389/frobt.2022.817870

**Published:** 2023-01-20

**Authors:** Simone Monteleone, Francesca Negrello, Giorgio Grioli, Manuel G. Catalano, Antonio Bicchi, Manolo Garabini

**Affiliations:** ^1^ Centro di Ricerca E. Piaggio e Dipartimento di Ingegneria dell’Informazione, Università di Pisa, Pisa, Italy; ^2^ Istituto Italiano di Tecnologia, Genova, Italy

**Keywords:** benchmarking method, self-stabilizing robots, robots balance, performance assessment, robustness

## Abstract

Robots that work in unstructured scenarios are often subjected to collisions with the environment or external agents. Accordingly, recently, researchers focused on designing robust and resilient systems. This work presents a framework that quantitatively assesses the balancing resilience of self-stabilizing robots subjected to external perturbations. Our proposed framework consists of a set of novel Performance Indicators (PIs), experimental protocols for the reliable and repeatable measurement of the PIs, and a novel testbed to execute the protocols. The design of the testbed, the control structure, the post-processing software, and all the documentation related to the performance indicators and protocols are provided as open-source material so that other institutions can replicate the system. As an example of the application of our method, we report a set of experimental tests on a two-wheeled humanoid robot, with an experimental campaign of more than 1100 tests. The investigation demonstrates high repeatability and efficacy in executing reliable and precise perturbations.

## 1 Introduction

The growing employment of robots in real-world applications, e.g., exploration of hazardous environments (Negrello et al., 2018) and household assistance (Parmiggiani et al., 2017), emphasizes the necessity of robots safe and resilient against disturbances. In engineering, Hollnagel et al. (2006) defined resilience as “the ability of an organization (system) to keep or recover quickly to a stable state, allowing it to continue operations during and after a major mishap or in the presence of continuous significant stresses”. Zhang brought the concept of resilience into the robotic field (Zhang and Lin, 2010), while Zhang et al., 2017 proposes a set of principles for the design of soft and resilient robots.

Following Hollnagel and Zhang’s interpretations, we investigate the definition of resilience for self-stabilizing robots. Self-stabilizing robots are a group of robotic systems with the common trait of possessing an unstable equilibrium stabilized continuously through control. Their increment of control and design complexity is accepted in the face of the augmented dexterity and agility that they show when compared to stable robots, such as mobile base robots (Fuchs et al., 2009). In the face of this augmented dexterity, the possibility of facing unexpected falls, which may cause damage to the robot, the surroundings, or persons, arises and become the major issue for a self-stabilizing robot to cease operations. As a result, for these robots, the concept of resilience expressed by Zhang and Lin, 2010 should be re-defined as the ability of a system to maintain a stable state, allowing it to continue operating in the presence of continuous and significant perturbations. In this sense, studying the resilience of robots becomes closely connected to looking into balancing abilities.

However, nowadays, the measurement of robots’ balancing resilience is a novel research field and still mostly relies on qualitative methods. To this aim, one of the most advanced fields is the legged locomotion research community. Nevertheless, in the related literature, it is possible to find just heuristic tests: Pushes) (Barasuol et al., 2013) (Feng et al., 2016), tilting the support surface (Li et al., 2013), balancing over a soft ground (Henze et al., 2016), or impacting with heavy masses (Kanzaki et al., 2005). These assessment methods are qualitative or hardly repeatable and do not allow comparisons between different robots. Benchmarking the performance of robotic systems offers many advantages. It allows for quantifying the performance of various systems, making comparisons possible, and fostering improvements. In industry, performance quantification makes possible standardization of technologies and regulation of the processes for manufacturing and commercialization of certified robots (Torricelli et al., 2015). Hence, a growing interest in the field of benchmarking has arisen during recent years in the research community (Negrello et al. (2020)) (Stasse et al. (2018)), especially for legged systems (Torricelli et al., 2015). Torricelli et al., 2015 and Torricelli and Pons, 2018 paved the way for benchmarking platforms for self-stabilizing robots and exoskeletons with the European Project EUROBENCH 2020^1^. Seventeen sub-projects work under the Eurobench consortium, each accounting for a different aspect of robot performance. To give some valuable examples, in Taborri et al., 2020, the authors present ***B.E.A.T.***, a benchmark for evaluating the static and dynamic balance of wearable human-assisting devices. In Lippi et al., 2019, Lippi et al., 2020, the authors proposed ***COMTEST***, a similar framework for testing the performance of humanoids, as well as a set of Performance Indicators that aim to standardize the capabilities of robots on a universal level. In Vicario et al., 2021, the authors present ***FORECAST***, a benchmarking method able to “define an objective score for a given force-controlled system accounting for its sensitivity to environmental uncertainties and variations.” Lastly, in Bayón et al., 2021, the authors proposed ***BenchBalance***, a.“ Benchmarking solution proposed to conduct reproducible assessments of balance in various conditions, mainly focused on wearable robots but also applicable to humanoids.”

We propose an evaluation framework for characterizing the resilience of self-stabilizing robots subjected to external disturbances (Monteleone et al., 2020). In Monteleone et al., 2020, we introduced the early conceptual definition of the testbed, with experimental validation on a two-wheeled robot solely on impulsive conditions, enforced *via* a non-actuated prototype of the testbed. In this work, we developed further the conceptual definition of the testbed, designing a framework composed of seven novel PIs to evaluate the resilience of a robot, five original experimental protocols for assessing the PIs, and a new testbed for reproducible issuing of both dynamic and static perturbations. The novel framework comprises an actuated structure equipped with a brake and clutch to perform various disturbances and protect the robot and operators against accidental impacts. The PIs, the experimental protocols, and the actuated and adjustable structure design are novelties in the state of the art. The proposed system draws inspiration from classical resilience testing machines used for the characterization of materials samples (as the Charpy test stand (ISO, 2010), realizing a system that can apply a desired impulsive, repetitive, or static disturbance in the most straightforward and easily reproducible way. As a previous work, we designed a non-actuated benchmark structure to test the resilience of the soft hand grasping under impulsive loads (Negrello et al., 2020). The novel system integrates position and force sensors to characterize the disturbance we are applying to the robot. It is actuated to control the application of perturbations under static and dynamic conditions. As a specific use case of applying our method, we report a set of experimental tests on a particolar two-wheeled base humanoid robot (Lentini et al., 2019). The main contributions of this work are the definition of the performance indicators, the testing protocols, and the mechanical design and control of the testbed. Additionally, all the materials are presented as open source and can be found on the external link in Section *“Data Availability Statement”*.

The resilience characterization framework we propose will pave the way for a rigorous benchmarking process of robot performance. The impact of our framework could go far beyond the balancing resilience characterization of wheeled robots (such as Alter-Ego from IIT/Research Center “E. Piaggio” or Golem Krang from Georgia Institute of Technology (Stilman et al., 2010) and could include the assessment of the balancing of autonomous legged robots. Today autonomous legged robotics is one of the most vibrant and hot research topics and is also significantly changing the industrial landscape. The population of humanoid (see, e.g., COMAN from “Istituto Italiano di Tecnologia (IIT)” (Tsagarakis et al., 2013) or Valkyrie from NASA (Radford et al., 2015) and quadrupedal (see, e.g., HyQ from IIT (Semini et al., 2011) or Anymal from ANYbotics (Hutter et al., 2016) prototypes has dramatically increased in the last decades, as well as the related scientific publications. Moreover, today there exist several companies that develop and commercialize legged robots (e.g., agilityrobotics^2^, unitree^3^, ANYbotics^4^, SoftBank^5^), with the remarkable recent acquisition of Boston Dynamics^6^ by a large automotive corporation, promising to be the core of a novel industrial segment. Finally, a strong impact of our work is expected in the field of assistive robotics (e.g., wearable robots (Kazerooni, 2005) (Khazoom et al., 2020) and personal robots (Parmiggiani et al., 2017), that is experiencing a growing trend similar to one of the autonomous legged robots.

The paper is organized as follows: Section 2 briefly recalls the requirements necessary to define a robotic system performance. Section 3 describes the performance indicators that we propose to define the resilience and performance of systems subjected to external perturbations. Section 4 and Section 5 present the design of the testbed, which we use to test the systems and the experimental protocols to execute reliable and repeatable experiments with different perturbation conditions. Section 6 shows the results of applying this testing strategy to Alter-Ego, a two-wheeled humanoid robot. Section 7 discusses the results based on the acquired data, exposing the quality and the possible future improvements of the benchmarking method. Section 8 concludes the paper.

## 2 Methodology and concept

Given the youth of robotics (about 60 years), benchmarking the performance of robots is a novel study area. Other fields, such as biomechanics, have extensively researched this problem to assess, for example, humans’ balance and locomotion capabilities. The literature proposes several methods and structures to characterize the performance of human balancing (e.g., Berg et al., 1992; Zemková, 2011; Molnar et al., 2018). They typically rely on the application of perturbations to the subject, e.g., asymmetric (Vashista et al., 2013), impulsive (Ellis et al., 2014), or active (Vashista et al., 2014) disturbances at the Center of Mass (CoM), or on the distal arts, e.g., Barasuol et al., 2013. From the literature, the importance of employing different forms of disturbances to measure the performance of a system is evident. To characterize the balancing performance of a robot, we require a system capable of providing a variety of perturbations on robotic structures and collecting a meaningful set of data.

When applied to robotic applications, these stimuli can be helpful in evaluating performance in typical stress situations. Impacts are disturbances that naturally occur in unstructured environments and collaboration with humans. Single impacts are the most common perturbations in robotics, but various types of pushes occur periodically or that last over time. Repetitive and quasi-static perturbations are sustained during Human-Robot Interactions (HRI). This kind of stimulus helps people perceive robots as human-like entities rather than mechanical systems (Hyon et al., 2007).

**FIGURE 1:**
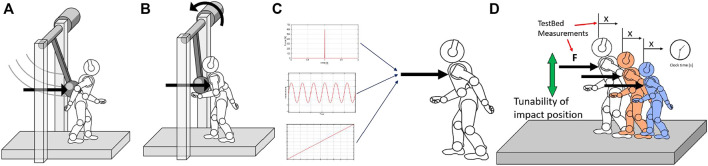
Conceptual design of the structure. **(A)** Represents the impulsive, highly dynamic experiments, while **(B)** represents the quasi-static experiments. **(C)** Definition of the required experiments for Performance Characterization. **(D)** Basic features of the testbed, to perform test on different robots. Panel **(A)** and **(B)** are taken from Monteleone et al. (2020), a previous work of the authors.

By analyzing the response of systems subjected to various types of disturbances (see Section 3), we point out the necessity to define some indices that can easily show the limits and capability of systems. In this way, we can determine the experiments necessary to obtain the desired performance indicators (Figure 1C) that will constitute the core of our protocols and structure. In the case of repetitive or quasi-static perturbation, we believe it is worth dividing the stimuli between position-driven and force-driven ones. Indeed, despite their superficial similarities, robots react to CoM position perturbations and force perturbations in distinct ways. Repetitive perturbations are the finest example (see Section 6).

Perturbations can be applied to robots they are while moving or standing still. Balancing in a standing position may appear a trivial task, but it is the primary condition for any self-stabilizing robot to work. Balancing in the presence of perturbations is a crucial topic in the literature. As examples, in Stephens, 2007, Ott et al., 2011, and Liu and Atkeson, 2009, the authors present strategies and controllers to recover from significant disturbances and maintain an upright posture. Understanding the limits of performance in robot balancing is the basis for fostering self-stabilizing system technologies.

During data acquisition, we must rely on measurements resulting from the testbed sensors. The test bench would be used to assess multiple robots, and we can not know *a priori* which measurements are accessible from the robot side. Accordingly, the measured values of the test bench sensors should be consistent, simple, and repeatable, allowing more reliable performance computations. Measures coming from robots are not sufficient, and therefore, we must rely only on commercial sensors integrated into the test bench. We want to investigate robotic structures that may have very different dimensions. Due to that, the test platform should adapt to the size of the robot under evaluation (Figure 1D). The test bench should be fully modular, allowing different disturbance conditions and locations. Complete system modularity guarantees the highest flexibility during tests. The use of the proposed device could be extended to other benchmarking scenarios, especially those involving stability against disturbances on different terrains, such as walking on slopes or irregular terrain.

During the development of the conceptual structure, we design a pendulum-like system, aiming to make its main dimensions (such as the height of the pendulum shaft, the length of the pendulum, and inertia) adjustable to match one of the different robots. In the design phase, we focus on the key features the benchmark must possess (Figure 1D). The primary purpose of this structure is to collect data from the robotic systems to define a set of Performance Indicators. These indicators are detailed in the next section.

## 3 Performance indicators

PIs describe the resilience of a self-stabilizing robot and allow comparisons among different robotics systems. The resilience of robots, in particular self-stabilizing systems, is influenced by structural robustness, but their balancing capabilities also cover a significant role. We divided PIs into two categories. The first contains those indices that show the limits at which the robot loses its balancing capability (see Section 3.1). The second is composed of those indices that describe the properties of systems subjected to perturbations (see Section 3.2).

### 3.1 Resilience limits

#### 3.1.1 Impulsive resilience

**FIGURE 2:**
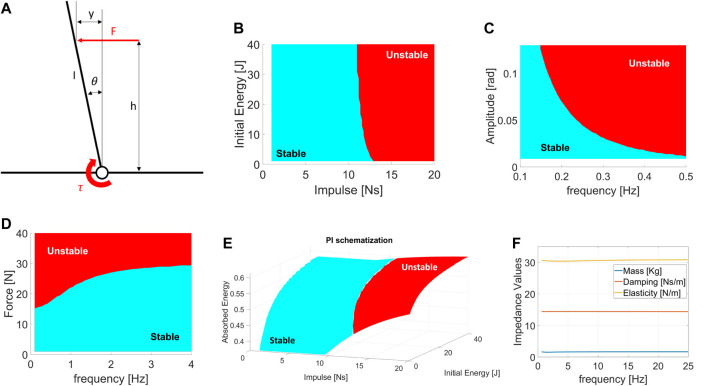
**(A)** Schematized Model used to visualize the Performance Indicators results. Example of: **(B)** Impulsive resilience chart. **(C,D)** Excited Resilience charts, where **(B)** is the ERrelated to a displacement perturbation, while **(C)** is the ERrelated to an oscillating force. **(E)** Absorbed Energy chart during Impacts. **(F)** Excited Equivalent Impedance chart. All picture refers to the model in **(A)**. The region of conditions at which the robot shows unstable behavior is reported in red.

Impulsive Resilience (IR) defines the maximum impulsive perturbation a robot can withstand without breaking or falling. Impacts are described by the impulse (*I*) and energy involved (*E*). Therefore, the IRis a diagram in which *I* lies on the x-axis and *E* on the y-axis. The resulting “*resilience regions*” (Figure 2B) are areas of the graph that describe the conditions at which the robot withstands the shock (light blue) or falls (red).

#### 3.1.2 Excited resilience

The Excited Resilience (ER) defines the maximum perturbation a robot subjected to repetitive shocks can tolerate without breaking or falling. A repetitive disturbance is described by its amplitude (*A*) and the frequency (*f*) at which it is repeated. More in detail, the load can be a displacement or force perturbation. Hence, the ERare two plots in which *f* lies on the x-axis and *A* on the y-axis. The first shows the resilience regions of the robot subjected to repetitive displacement oscillations (Figure 2C), while the second displays the resilience regions related to force oscillations (Figure 2D).

#### 3.1.3 Quasi-static resilience

The Quasi-Static Resilience (QSR) defines the maximum perturbation a robot subjected to constant loads tolerates without breaking or falling. A constant load is described by its value in terms of force or displacement. The QSRcomprehends the minimum unstabilizing constant force and displacement measures. Hence, QSRresults in two scalar values. Note that in the case of a robot that can perform balancing actions, such as backward steps, these values are converted to the minimum force and displacement that induces the system to perform a complex balancing routine.

### 3.2 Robot properties

#### 3.2.1 Absorbed energy

The Absorbed Energy during Impulsive perturbations (AEI) defines the capability of a robot to absorb energy during impacts. The AEIindicates the capability of the robot to oppose an impact and is expressed by the percentage of energy absorbed. Being a PI related to impulsive shocks, the parameters that describe the AEIare *I* and *E*. The result is a three-dimensional plot in which *I* lies on the x-axis, *E* is on the y-axis, and the percentage of energy absorbed by the robot is on the z-axis (Figure 2E).

#### 3.2.2 Excited equivalent impedance

The Excited Equivalent Impedance (EEI) evaluates the dynamic behavior of a robot when subjected to repetitive disturbances. The EEIconsiders a simplified standard model and computes the dynamic coefficients of inertia (*J*), elasticity (*K*), and damping (*B*), varying *f*. The estimations of these parameters rely on the measurements from repetitive force perturbations. Using a dynamic regressor, we compare the robot to a second-order inertia-spring-damper system and evaluate the coefficients [*J*, *B*, *K*]. The EEIis a plot with *f* on the x-axis and the impedance coefficients on the y-axis (Figure 2F).

#### 3.2.3 Normalization factor

Performance Indicators describe the balancing skills of systems under different loads. PIs are expressed by extensive measures (such as forces and displacements); therefore, these indices are highly dependent on the robot’s size. As a result, to compare different systems, it is necessary to scale all measurements to a common reference model. Any tested robot could be used as a standard for all other systems. However, we believe it is better to refer to a more general model.

Since robots are designed to mimic human behaviors, we compare their performance to a medium-sized human. Dimensions for the human model are retrieved from Armstrong, 1988. Using these values as standard dimensions, we designed some normalization factors that weigh all the previous indicators on the common model.

**FIGURE 3:**
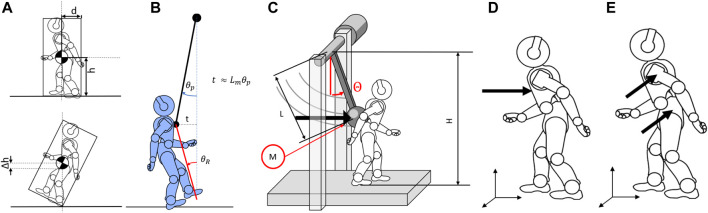
Schematic visualization of the model for **(A)** the force and energy normalization factor computation and **(B)** the displacement normalization factor. **(C)** Model of the structure reporting the main dimensions of the system. **(D,E)** Definition of the impact position for **(B)** frontal perturbations and **(C)** sagittal perturbations.

We define a total of six Normalization Factors: two are related to force scaling (i.e., frontal and lateral directions), two are related to the energy scaling (as before, frontal and lateral), and the last two are related to the CoM displacement. Normalization factors for energy $\left(N_{E_{J}}\right)$ and force $\left(N_{F_{J}}\right)$ have been calculated considering the systems rigid and approximable to parallelepipeds (Figure 3A). The force and energy normalization factors are computed as the minimum force and kinetic energy required to unstabilize the system by pivoting around one of its edges (see Figure 3A). Hereafter, we compare these values to the ones of our reference system. The normalization factors related to the force are

$$N_{F_{J}}=\frac{m_{\mathrm{robot}}}{m_{\mathrm{human}}}\frac{d_{CoM_{\mathrm{robot}}}}{d_{CoM_{\mathrm{human}}}}\frac{h_{CoM_{\mathrm{human}}}}{h_{CoM_{\mathrm{robot}}}},$$ (1)

while the normalization factors related to the energy are

$$N_{E_{J}}=\frac{m_{\mathrm{robot}}}{m_{\mathrm{human}}}\frac{\sqrt{h_{CoM_{\mathrm{robot}}}^{2}-d_{CoM_{\mathrm{robot}}}^{2}}-h_{CoM_{\mathrm{robot}}}}{\sqrt{h_{CoM_{\mathrm{human}}}^{2}-d_{CoM_{\mathrm{human}}}^{2}}-h_{CoM_{\mathrm{human}}}}.$$ (2)

For both equations, *m*_*i*_ are the masses of the robot and human, $h_{CoM_{i}}$ are the height of the Center of Mass from the ground, and $d_{CoM_{i}}$ are the width of the bearing surface. The $d_{CoM_{i}}$ dimension is different if the normalization factor is computed frontally or laterally. Subscript *J* indicates that the same equation holds both directions. Normalization factors are calculated by the minimum impulsive force and energy (respectively) that unstabilizes the model by pivoting it on its edge. Lastly, the normalization factor related to the displacement (*N*_*D*_) compares the angular movement of the contact point relative to the robot ground, scaling it to the movement the system would have with the dimensions of a medium-sized person (Figure 3B).

$$N_{D}=\frac{h_{CoM_{\mathrm{robot}}}}{h_{CoM_{\mathrm{human}}}}$$ (3)

### 3.3 Performance indicators illustration

In this paragraph, we aim to illustrate the PIs behavior when applied to a generic robotic system. Functional to the visualization of the PIs is a dynamical examination of an actuated inverted pendulum subjected to external perturbations (Figure 2A). This example model is chosen because it resembles the dynamics of a humanoid-legged robotic system subjected to pushes when no steps are performed, allowing us to consider the feet/base of the robot fixed on the ground. Therefore, this model is similar to a basic self-balancing robot performing *“ankle strategy”* (Stephens, 2007; Rogers and Mille, 2018). Different models can be applied if we aim to resemble two-wheeled humanoid robots, which typically act as cart poles. The reader should note that the choice of the model does not influence the effectiveness of the PIs, but it may vary the behavior shown.

The model dynamics for the inverted pendulum is

$$J\ddot{\theta }+Mg\frac{l}{2}\sin\left(\theta \right)=\tau -Fh,\;with\;\tau =-K_{\mathrm{lqr}}\left[\theta ;\dot{\theta }\right],$$ (4)

where *J*, *M* and *l* are the inertia, the mass, and the length of the inverse pendulum, respectively. *τ* is the commanded torque used to apply an LQR optimal control *K*_*lqr*_. Lastly, *F* is the external force, and *h* is the height at which we apply the perturbation. During simulations, we adjust the input function *F*(*t*) to match the different types of desired loads. Hence, the conditions under which a robot falls indicate the system’s resilience. In our model, we define the falling condition as the angle *θ* exceeding the limit *θ*_*max*_. Saturation of the maximum torque makes the systems more similar to real robotic systems.

### 3.4 Robot resilience datasheet

Table 1 presents a datasheet that we propose to summarize the balancing performance of a robot. We hope that a datasheet can be a helpful add-on to foster system comparisons and regulation of processes.

**TABLE 1:** Example of datasheet containing the resilience benchmark results.

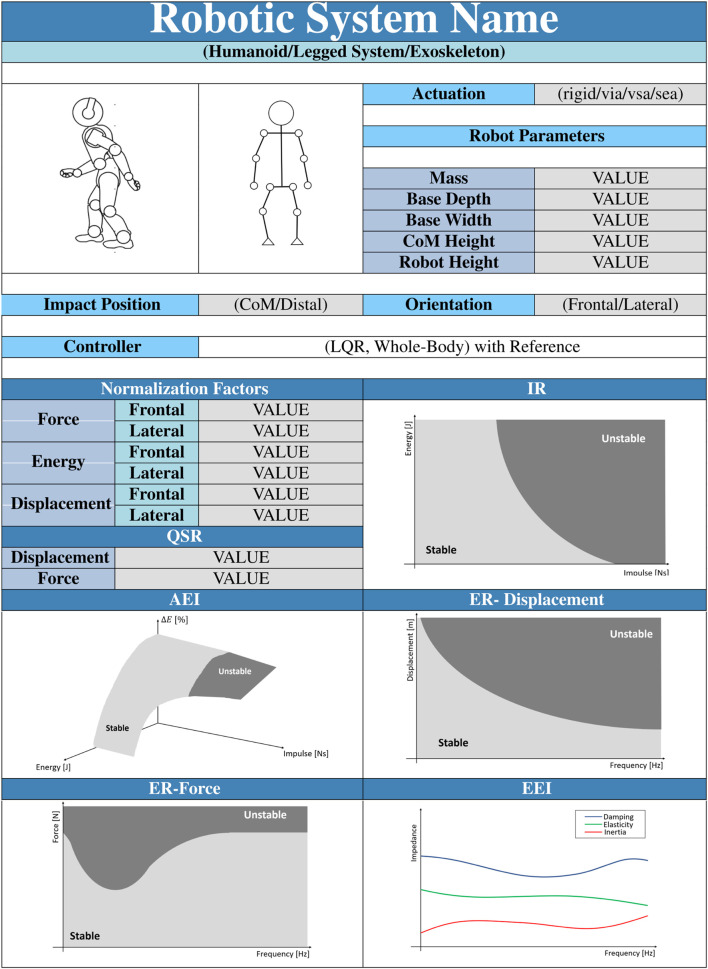

The datasheet is organized as follows. The first and second lines contain the name and the type of the robot (e.g., legged humanoids, quadrupeds, exoskeletons) under testing. The successive 2 cells show a photo of the system and a scheme of its kinematics. The cell “*Actuation*” defines which types of actuators the system is built with (actuation units can be rigid, SEA, VIA, etcetera (Vanderborght et al., 2013). “*Robot Parameters*” provides the main dimensions of the robot used to define the normalization Factors reported in the related cells. “*Impact Position*” and “*Orientation*” define the experimental conditions at which the experiments are executed. *Impact Position* describes the contact point location, while *Orientation* indicates if the PIs are related to the frontal or lateral perturbation on the robot. *Controller* means which control is applied to the robot, and a reference on the related paper is strongly recommended. The other cells (QSR, IR, AEI, ER-Displacement, ER-Force, and EEI) report the PIs of the robot under the described conditions.

## 4 Test-bench design and characterization

The definition of the PIs and testing conditions provides a set of characteristics the system must possess. In Monteleone et al., 2020, we introduce the early concept definition, focusing on five design features: flexibility, reproducibility, adjustability, indipendency as a system, and operator safety.

### 4.1 Mechatronic design

**FIGURE 4:**
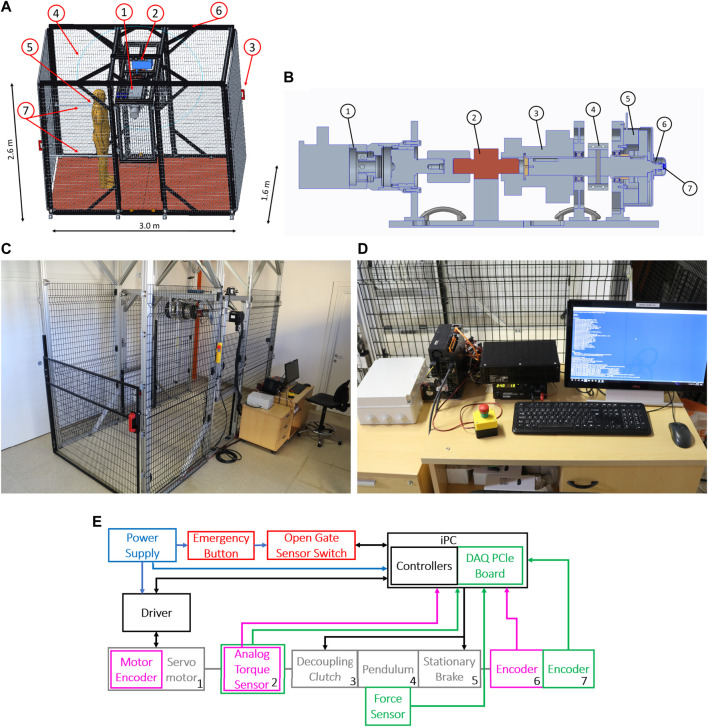
Photo of **(A)** Test-Bench dimensions and components: 1) Actuation group, 2) electrical crane, 3) sensorized safety doors, 4) safety panels, 5) agent positions during tests, 6) external structure, and 7) handrails. **(B)** Cross section of the actuation unit: From left to right, 1) servomotor, 2) torque sensor, 3) electro-magnetic clutch, 4) pendulum connector, 5) electro-magnetic brake, 6) first position sensor (absolute), 7) second position sensor (relative). **(C)** Testbed, and **(D)** industrial PC and controllers. **(E)** Electronic Connection of the Test-Bench. Blue arrows indicate the power connections, black arrows are the control communication network, and purple and green arrows describe sensor connections for the control loop and the data record. In red, we indicate the safety systems.

Figure 4A shows the structure. The testbed is composed of two parts. The first is the central span, consisting of the actuation group and the pendulum. The second part is the external structure, mainly composed of aluminum extrusions and safety nets.

Figure 4B shows the cross-section of the actuation unit and its main components. From left to right, there is a servomotor (1), a torque sensor (2), an electromagnetic clutch (3), the pendulum connector (4), an electromagnetic brake (5), and two absolute position sensors (6–7). In component 4, we mounted the pendulum bar. We placed a piezoelectric sensor on its tip to measure the contact force between the actuation group and the robot under test. To prevent misalignments of the torque sensor, we connect it to the actuator through an Oldham joint and to the clutch through an elastic component. The compliant joint also absorbs accidental shocks transmitted to the torque sensors.

During impulsive tests, the clutch safeguards the servomotor and torque sensors. It disengages the pendulum shaft from such delicate parts, preventing the transmission of shocks. After an impact, the brake permits the system to attenuate oscillations. It also improves testbed safety by halting the pendulum in the case of emergencies.

The external structure protects the operators during test execution. We enclosed the test platform in an area accessible by two doors and surrounded by safety panels. Doors equip two electromagnetic locks. As a result, the control system can detect the status of gates (open or closed) and lock them, limiting access to the experimental area when the pendulum is moving. If the doors are unexpectedly released, the system activates the brake while simultaneously disengaging the clutch, preventing the pendulum from moving while safeguarding the motor.

Moreover, we provide an emergency button, which stops the pendulum movement in the same way as if the door opened. Figure 4C shows a picture of the physical structure, also displaying the placement of the control system (Figure 4D).

To enhance tunability, the system is equipped with components that can vary its structural dimensions. The testbed is provided with an electrical crane and four guides to change the position of the central span. Hence, it is possible to modify the height at which the pendulum impacts the robot (*H*). Furthermore, a two-part connector links the pendulum bar to the shaft, making it simple to vary the pendulum length (*L*). Lastly, we ensure that additional masses (*M*) can be mounted on the pendulum to increase its inertia. Table 2 reports the tunable parameters and their range of variation. We equipped the testbed with a modular floor with a series of holes equidistant from each other. This design improves the structure compatibility with other testing devices, such as treadmills or inclined planes. It also allows for the placement of obstacles to test robotic systems on uneven terrains.

**TABLE 2:** Test-bench characterization.

Tuning parameters	Range	Steps	
Pendulum bar length	.5 ÷ 1.5 *m*	0.5 *m*	
Pendulum axis height	1 ÷ 2 *m*	1 *cm*	
Additional masses	0 ÷ 15 *kg*	0.5 *kg*	
pendulum position	−90° ÷ 0°	.5°	
Friction Experimental Estimation			
	Pendulum length		
	0.5 m	1.0 m	1.4 m
Friction torque (*τ*_*f*_)	2.3 *Nm*		
Impulsive tests (Protocol 1)			
Maximum force	1200 N		
sinusoidal tests (Protocol 2/3)			
Maximum force	700 *N*	350 *N*	250 *Nm*
Maximum torque	350 *Nm*		
Maximum angular speed	100 *rpm*		
Maximum oscillation frequency	7 *Hz*	5 *Hz*	3 *Hz*
quasi-static tests (Protocol 4/5)			
Maximum force	700 *N*	350 *N*	250 *Nm*
Maximum torque	350 *Nm*		
Maximum angular speed	100 *rpm*		
sensors resolutions			
torque sensor force resolution	1 *N*	0.5 *N*	0.4 *N*
force sensor resolution	0.8 *N*		
angular encoder resolution	.09^*o*^		

Table 2 reports the main characteristic values of the testbed. Friction torque has been computed experimentally.

### 4.2 Control architecture

The framework is equipped with an industrial PC that is ROS compatible and three drivers for the servomotor, clutch, and brake, respectively. The IPC supervises the structure framework, generating the control inputs that are communicated to drivers. Moreover, it also acquires data through an integrated DAQ system from National Instruments (NI) (Figure 4D).

Figure 4E describes the control architecture scheme, showing each block and its physical connections. For the actuation unit components, each number corresponds to the ones shown in Figure 4B. Blue arrows indicate the power connections, black arrows show the control communication network, and purple and green arrows depict the sensor connections for the control loop and the data record, respectively.

The actuation unit can be controlled both in position or torque loop. The position control loop uses the measurements of the Renishaw absolute encoder (6) located at the output shaft. However, if the clutch is disengaged, the system relies on the servomotor encoder to move the actuator and reset the zero position of the motor control. The torque control loop relies on the measurement of the FUTEK torque sensor (2). These measurements are corrected by gravity compensation, so if we command a force trajectory, the torque measures reject the pendulum weight, providing the correct movement at the contact point.

### 4.3 Data recording

The IPC saves data from experiments through a National Instrument data acquisition device. We acquire data from three sensors. The first is the pendulum encoder (number 7 in Figures 4B, E), which is an AMS absolute encoder with a resolution of about .1°. The second is the force sensor. It is a DYTRAN 1051V6, a piezoelectric sensor capable of precisely measuring impacts and impulsive forces. However, when subjected to constant or slowly varying forces, it does not perform correctly due to drift. It has a resolution of 0.3 *N* and a saturating value of 2224 *N*. The third one is the torque sensor. It is a FUTEK FSH02060, an analog sensor that measures non-impulsive forces using strain gauge technologies. The resolution of the torque sensor is 1 *Nm*, and its maximum measurable value is 500 *Nm*. We use the force sensor during impulsive tests to compute performance indicators. In contrast, during the other tests, we estimate the force exerted on the pendulum, knowing the distance of the pendulum tip to the torque sensor axis, and correcting the measure with a gravity compensation in post-processing.

The testbed acquires all data at a frequency of 10 kHz. Position and torque measurements are filtered by excluding outliers and using a symmetric moving average filter. The data from the piezoelectric sensor is not filtered because filtering would result in a loss of accuracy on the force peaks. From the force data, it is possible to identify the exact moment of an impact. However, to measure the value of the impulse, there exist two methods. The first technique estimates the duration of the contact between the robot and the pendulum and integrates the force value. The second method relies on measuring the pendulum velocities before and after the moment of touch. It evaluates the impulse as the variation of the momentum. We saw experimentally that the second one resulted in being more reliable, as the definition of the contact duration is not trivial.

## 5 Experimental protocols

To measure the PIs, we developed a series of testing methods that allow the reproduction of the necessary perturbations. In the following, we define each protocol and report the detailed procedure to perform the experiments. This work focused on the definition of resilience against pushes on regular, obstacle-free terrains. The possibility of studying the effects of different terrains on the performance is left to future works.

During a protocol execution, we repeat each experiment (we call “experiment” tests with the same set of conditions) 10 times (we call each one a “run”). With this, we aim to provide the results with a certain degree of statistical validity. Indeed, we performed a high number of experiments to provide a more reliable view of how the system reacts to perturbation with a given entity. Since the system is physical, borderline values of perturbation can lead to a robot falling or not depending on other robot conditions (e.g., if the robot is impacted while the pitch angle is positive or negative). Therefore, the high number of experiments considers the fall’s statistical validity, reducing the effect of outlier situations.

We measure the pendulum angle, the torque at the motor axis, and the force at the contact point with the robot. These measures are used to obtain all the performance indicators in Section 3. The force sensor employs piezo-electric technologies, allowing one to appreciate the quick variation of forces, such as peaks. On the contrary, since the torque sensor is resistive, it is more suitable to evaluate constant or slow-vary forces.

At the beginning of each protocol execution, we must adjust the structure to impact the system at the desired contact point. For frontal collisions, the designated point should be placed at the center of the chest, on the robot axis, the closest to the CoM as possible (Figure 3D). The height of the contact point is measured and saved by the platform. For side impacts, the contact point should be located on the shoulder or hip, typical contact points during accidental collisions (Figure 3E). Aside from the contact point, lateral experiments execution uses the same experimental protocols as the frontal experiment. Therefore, the following section will not further distinguish between frontal or lateral protocols. The control sets the end of the experiment when it detects that the pendulum has reached the maximum height and there has been a speed inversion or when it is motionless.

### 5.1 Protocol I: Impulsive disturbance protocol

The first protocol aims to assess the balancing performance of systems subjected to impacts. Impulsive loads are obtained by raising the pendulum at the desired height and successively letting it free to fall.

In the Charpy test, impacts are defined by the energy involved in the experiment (François and Pineau, 2002). Moreover, impacts can always be described by the value of forces or impulses exchanged between objects (Stronge, 2018). Hence, we decided to define impulsive tests based on the value of both impulse (*I*) and the initial energy (*E*). We discovered that these parameters could be treated as two independent values through analytical computations and experiments. To obtain the desired values during experiments, we tune the pendulum length (*L*), the initial position (*θ*), and pendulum inertia (*M*) (see Figure 3C). We related the pendulum parameters with potential energy and the impulse exerted on the system during an impact. Parameters of impulsive tests are defined by

$$\left\{\begin{array}{c} E=\left(M+\frac{\delta L}{2}\right)gL\left(1-\text{cos}\;\theta \right)\\ I=\frac{1}{L}\sqrt{2\left(ML^{2}+\frac{\delta L^{3}}{3}\right)\alpha E}\\ \alpha =1-\frac{\tau_{f}\theta }{E}; \end{array}\right..$$ (5)

Among them, *α* indicates the percentage of energy not lost due to friction and is computed experimentally by estimating the energy loss between initial height and impact height. *τ*_*f*_ represents the friction torque, which is assumed to be constant. *δ* expresses the linear density of the pendulum bar, and *g* is the gravity coefficient. These equations consider that the pendulum stops after the impact and that the impact occurs between rigid bodies, so there is a slight difference between theoretical and experimental values. The experimental procedure for the protocol I is reported in Table 3. It is worth noticing that *H*, *L*, and *M* change in response to the values of [*E*, *I*], whereas the others have a fixed value for each robot under test.

**TABLE 3:** The table shows the procedures for the testing protocols. If the steps are different, we divided it for each protocol.

Protocols procedure			
Steps	Protocol I	Protocol II *&* III	Protocol IV *&* V
1	Set up *H*, *L*, and *M*	Set up *H* and *L*	
2	Place the agent at the desired experiment position		
3	Activate the protocol		
4	Pendulum raises at desired position	Pendulum reaches contact point with the robot	
5	Data acquisition is started		
6	Pendulum performs the desired perturbation		
7	When experiment finishes, data acquisition stops and the pendulum moves to a resting position		
8	Operator reports if the robot is fallen, so that data can be saved		

**TABLE 4:** Datasheet resulting from the experiments on AlterEgo.

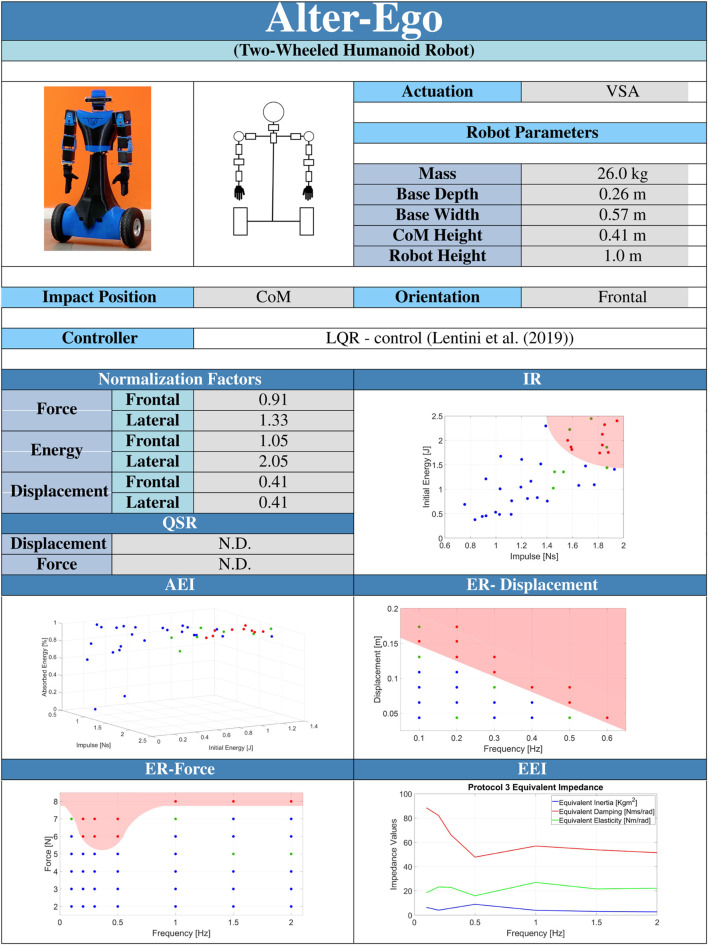

The medium execution time for each run is 2/3 s. The data collected during the experiments are used to compute the Performance Indicators described in Section 3. In particular, this protocol aims to find the IRand the AEI.

Defining the parameters that describe an impact requires a preliminary testing phase on a mock model. The mock model comprises an inverted pendulum structure and a small base to stabilize it. Following an impact, we let the system fall to reduce the residual noise on the force sensor.

**FIGURE 5:**
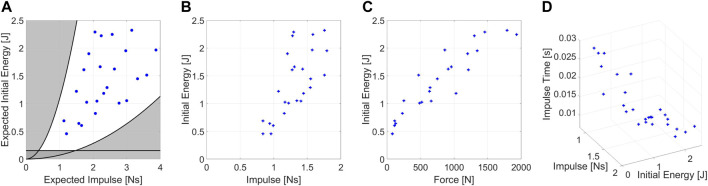
Experimental condition of the mock model tests. **(A)** Expected values of impulse and energy for the impacts on mock model, **(B)** real values of impulse and energy during test. **(C)** Forces resulting from the experiments. **(D)** Medium impulse time of the experiments depending on the test conditions.

As a preliminary couple of parameters, we selected the energy and the maximum exerted force ([*E*, *F*_*max*_]), assuming an impact lasted for a constant time. However, the assumptions resulted in being incorrect. Indeed, we experimentally observed that *E* influenced the impact duration (Figure 5D) and, consequently, *F*_*max*_ (Figure 5C). Conversely, the experiments show no correlation between *E* and the impulse (*I*) applied to the robot. Figure 5A, B show the theoretical values of *I* and *E* computed using Eq. 5 and the one resulting from experiments. We appreciate how assumptions about shocks (rigid impulse and stationary pendulum after impacts) generate a plot in which *I* values are scaled by a medium scale factor of .59.

Figure 5A highlights in black the physical constraints of the system. Points outside the demarcated area are not feasible due to the range of possible pendulum inertia.

Moreover, values below the straight line are not recommended because the friction action consumes most of the energy during the pendulum swing.

### 5.2 Protocols II *&* III: Sinusoidal protocols

The purpose of the second and third protocols is to assess the balancing performance of systems subjected to periodic perturbations. Repetitive perturbation are given with a controllable position amplitude (*A*_*D*_ for displacements, *A*_*F*_ for forces), frequency (*f*), and number of cycles (*n*_*C*_) in the form

$$\begin{array}{c} D\left(t\right)=A_{c}+A_{D}+A_{D}\text{sin}\left(2\pi ft-\pi /2\right),\\ F\left(t\right)=F_{c}+A_{F}+A_{F}\text{sin}\left(2\pi ft-\pi /2\right) \end{array}$$ (6)

where *A*_*c*_ is the position in which the pendulum starts to contact the robot, and *t* is the time. *A*_*F*_ is the force amplitude, and *F*_*c*_ is a small force to ensure that the robot will keep contact during the execution of the experiment. The first equation is related to protocol II, while the second one is to Protocol III. A position perturbation does not ensure that the contact lasts during all the experiment execution since we command the position of the pendulum to follow a specified path.

Table 3 reports the experimental procedure for protocol II *&* III. For Protocol II, the operator must stop the experiment if the robot falls. In contrast, for protocol III, if the robot falls, the framework will detect that contact with the robot is lost and consider the experiment finished. Experimental conditions ([*A*_*D*_, *f*] for protocol II, [*A*_*F*_, *f*] for protocol III) are gradually increased until the robot falls. The execution time heavily depends on the *f* at which the experiment is executed. The result of sinusoidal protocols is the ERof the system. Moreover, sinusoidal force protocol aims to define also the EEI, since the contact lasts along all the run execution. The main limitations are the maximum allowable frequency and amplitude during test execution. The maximum force and frequency depend on the characteristic of the actuation unit. The maximum displacement is a function of *f* (as it is related to the maximum allowable speed at the servomotor side) but also depends on the dimension of the robot. Indeed, since a displacement along the perpendicular direction corresponds to a height variation, the contact point should never exceed a safe height variation to avoid the system impacting sensitive parts of the robot, such as the head.

### 5.3 Protocol IV *&* V: Quasi-static protocols

The fourth and fifth protocols aim to assess the resilience of systems subjected to constant or quasi-static perturbations. We provide slow varying perturbation *D*(*t*) and *F*(*t*), with a dynamics of

$$\begin{array}{c} D\left(t\right)=A_{c}+V_{d}t,\\ F\left(t\right)=F_{c}+V_{f}t \end{array}$$ (7)

where *V*_*d*_ and *V*_*f*_ are the small velocity at which we execute the experiments. Tests are defined based on the value of *V*_*d*_ and *V*_*f*_ at which experiments are executed. However, the slopes of ramps provided are fixed to avoid testing the robot under non-quasi-static conditions.

Table 3 reports the experimental procedure for protocol IV *&* V. The falling detection algorithm is also applied in the case of quasi-static protocols. The falling detection algorithm explained in protocol III is also applied for protocols IV and V since the slow slopes ensure that contact is always present. Forth and fifth protocols are designed to measure the QSR. The major constraint is the maximum allowable displacement the system can do. The variation of height must be limited so that sensitive parts of the robot are kept safe. The possibility of studying the effects of different terrains on performance is left for future works.

## 6 Application example

To demonstrate the strength of our framework, we benchmark the performance of Alter-Ego, a robust and versatile mobile two-wheeled system with a functional anthropomorphic upper body (Lentini et al., 2019). The robot is equipped with an LQR optimal controller for lower body stability, while the upper body is controlled to stay in the rest position with a low level of stiffness. The robot is equipped with an integrated safety system (Zambella et al., 2020) that avoids breakages in case of falls. In the paper, we also report conditions at which the robot becomes unstable.

Tests are made on the frontal plane of the robot. Every test has the same contact point in the center of Alter-Ego chest, at the height of 80 *cm* from the ground. As stated in Section 5, experiments stop if the displacement of the pendulum exceeds the maximum allowable for the robot. The maximum permissible displacement of Alter-Ego was experimentally selected as 40 *cm* with a pendulum of 1 *m* length, corresponding to a height displacement of the contact point of around 8 *cm*. If necessary, the height displacement can be reduced using the 1.5 *m* pendulum bar.

Lateral experiments are not reported. The reason lies in the kinematics of AlterEgo that does not allow it to move laterally. No control can be applied in that direction. As a result, while performing Impulsive perturbations, the system acted rigidly until the impact was powerful enough to break the system. The robot resisted the external perturbation in the other protocols until the force was sufficient to lift the system. However, because we can easily calculate that force value analytically, we believed that a physical evaluation was unnecessary and would be detrimental to the robot integrity. Therefore, we decided to interrupt the lateral performance evaluation since continuously damaging the robot would have been too expensive.

We collect a number of 410 runs (41 different conditions) for protocol 1, 260 runs (26 different conditions) for protocol 2, 430 runs (43 different conditions) for protocol 3, and 10 runs each for protocols 4 and 5 for a total of 1120 experiments. A full testing procedure required around 4 days for frontal experiments. In all the figures related to performance indicators, we indicated with blue dots the conditions at which the robot does not fall, with green points conditions at which the robot falls beneath 30% of times, and with red dots conditions at which the robot falls with a statistical percentage above 30%. Table 4 reports the results of the experiment in the form of datasheet, as presented in Section 3.

In the attachment to the paper, we present a video showing examples of the execution of tests.

### 6.1 Protocol I: Impulsive protocol

**FIGURE 6:**
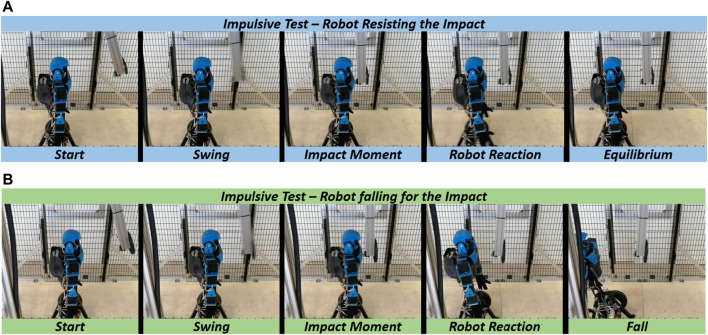
Photosequence of impulsive tests for **(A)** robot withstanding the impact, and **(B)** robot falling due to the impulse.

**FIGURE 7:**
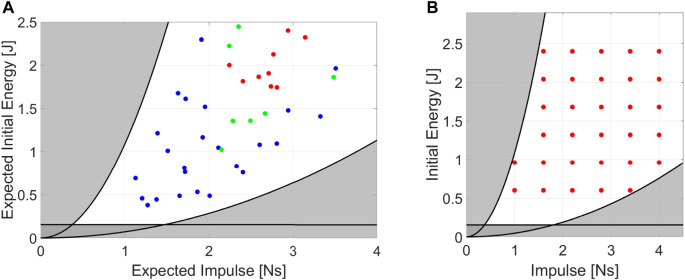
**(A)** Set of desired conditions at which we perform the experiments on Alter-Ego. Each point represents a set of [*E*, *I*] related to 10 runs. **(B)** Standardization on the selection of the impulsive experiments.

Figure 6 displays the photo-sequences of two experiments performed on Alter-Ego. The first one shows the system resisting an impact (Figure 6A), while the second shows the system failing to balance itself (Figure 6B). Impacts on the systems result in a variation in the pitch of the robot. If the pitch variation is too fast or too extended, the system cannot balance itself, failing. Tests on Alter-Ego have been executed with the conditions shown in Figure 7A. Each set of conditions ([*E*, *I*]) corresponds to a specific value of [*L*, *M*, *θ*_*i*_] for the experiment. To test the system, we performed the protocol described in Section 5.

**FIGURE 8:**
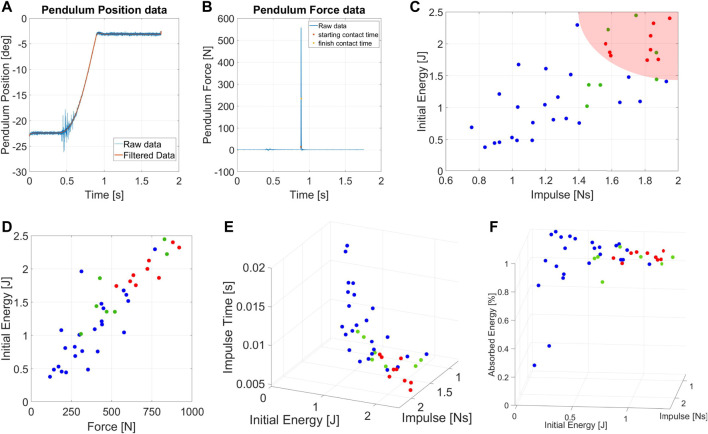
Measurement of **(A)** angular position and **(B)** force sensor data for the impulsive experiments. **(C)** Chart of the IRfor the Humanoid robot Alter-Ego. **(D)** Forces resulting from the impulsive tests applied Alter-Ego. **(E)** Impulse Time of the experiments varying the initial conditions. **(F)** Chart of the AEIfor the Humanoid robot Alter-Ego.

Figure 8 shows an example of angular position and force measurement during an impulsive test. Blue data indicates the raw data coming directly from the DAQ system, while we highlighted the filtered data in orange.

Figures 7C, F shows the IRand the AEIof Alter-Ego, respectively. AEIshows the capability of the robot to return energy to the pendulum in case of impacts with low [*E*, *I*]. In unstabilizing impacts, however, the robots absorb most of the energy, which becomes kinetic energy and plastic deformation of the covers. Figure 7C shows that it is possible to describe a region of conditions at which the robot cannot absorb and withstand the shock, validating our theory. In this graph, each set of data collected have a medium standard deviation from the mean value shown of around .37Ns, and therefore possess a certain degree of repeatability. Figure 7D reports the relation between *E* and *F*_max_ during impact. The graph shows an almost linear relation between those parameters, confirming the same results achieved with the mock model. Figure 7E shows the relation between impact conditions and impulse duration. This picture also confirmed the behavior exhibited by the mock model. Although it may seem obvious, this behavior is worth reporting. Indeed, the fact that Alter-Ego possesses more complex internal dynamics than the mock model does not change the considerations made about force and impulse time. Then, we can assume that this behavior holds for other robots, validating the choice of [*E*, *I*] as describing parameters for the experiment.

### 6.2 Protocol II: Sinusoidal displacement protocol

We began the experiments with the set of conditions [*A*_*D*_, *f*] = [4 *cm*, 0.1 *Hz*], gradually increasing them in ranges that goes from 4 *cm* to 17 *cm* within 7 steps for *A*_*D*_, and from 0.1 *Hz* to 0.6 *Hz* within 6 steps for *f*.

**FIGURE 9:**
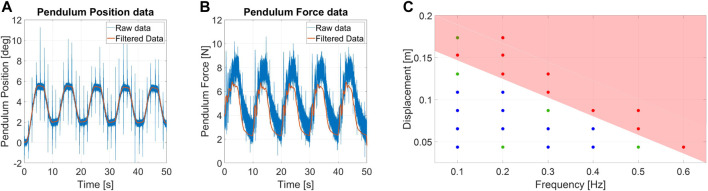
Measurement of **(A)** angular position and **(B)** force estimation for a sinusoidal displacement disturbance experiment. The used condition for the test are *f* = 0.1 *Hz* and *A*_*D*_ = 4 *cm*. **(C)** Chart of the ERrelated to displacement perturbations for Alter-Ego.

Figure 9 shows an example of measurements for sinusoidal displacement perturbations. Both position and force measurements required filtering noise and outliers. The reason behind the raw data drift lies in the absence of gravity compensation, which is adjusted during filtering. Figure 9C shows the ERrelated to displacement perturbations of the robots. The mean standard deviation that those data possess from their related medium value is 0.2 *mm*.

ERdepicts the relationship between the system capability to resist recurrent disturbances to their oscillation frequency. Higher frequencies in the position perturbation domain correspond to faster movements of the systems. Rapid perturbations result in being more unstabilizing than large displacements.

Analysis of the measurements deriving from this protocol shows a high degree of repeatability on the experimental conditions. Moreover, the procedure defined in Section 5 resulted in being simple and straightforward.

### 6.3 Protocol III: Sinusoidal force protocol

We began the tests with the set of conditions [*A*_*F*_, *f*] = [2*N*, 0.1*Hz*], gradually increasing them with steps of 1*N* for *A*_*F*_, and with a span of *f* = [.1, .2, .3, .5, 1.0, 1.5, 2.0]. Experiments are performed with a *n*_*C*_ = 5.

**FIGURE 10:**
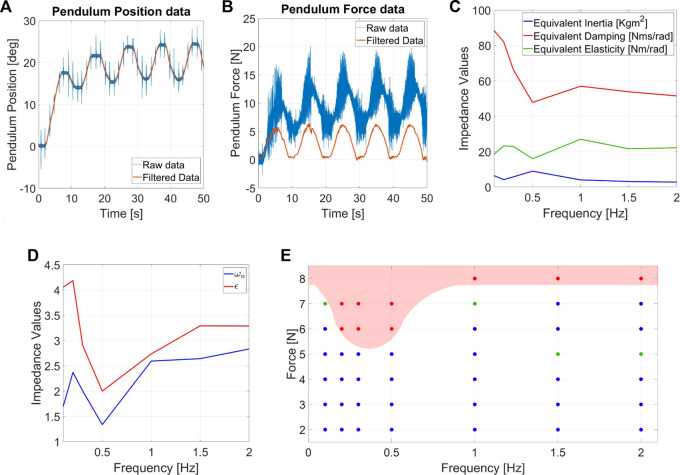
Measurement of **(A)** angular position and **(B)** force estimation for the sinusoidal force disturbance experiments. The used condition for the test are *f* = 0.1*Hz* and *A*_*F*_ = 3*N*. **(C)** EEIand **(D)** Equivalent damping ratio (*ϵ*) and natural frequency (*ω*_*n*_) related to sinusoidal force protocols. **(E)** Chart of the ERrelated to repetitive forces for Alter-Ego.

Figure 10 shows a set of measurements for sinusoidal force perturbation. During filtering of data, we took into account the effect of gravity on the torque measurements, and we compensated it to obtain the force exchanged between the structure and the robot.

Figure 10E shows the ERof Alter-Ego, while Figure 10C describes the EEI. For this set of data, the mean standard deviation is around 0.4*N* from their meadium value. The system is approximated to a second-order system (mass-spring-damper, see Section 3), and the equivalent coefficients are computed for each frequency.

ERreveals a low-frequency force range which is more destabilizing for Alter-Ego. Its dynamics act as a low pass filter, better rejecting high-frequency perturbations. In case of repetitive displacement perturbations, the pendulum provides faster and stronger pushes at higher frequencies, resulting in the robot that eventually falls when the frequency exceeds a definite value. Conversely, in the case of repetitive force perturbations, pushes act more like vibrations than perturbations at increasing frequencies, resulting in the robot rejecting these disturbances better than at lower frequencies. EEIshows a system with almost constant inertia and elasticity while the damping lowers at higher frequencies. Being the impedance an extrinsic property, we also computed the damping ratio and the natural frequency, which are intrinsic properties instead (Figure 10D). Interestingly, a reduction in the damping ratio and natural frequency occurs in frequencies that are more destabilizing for the ER.

Experiments demonstrate the repeatability of testing conditions and point out how the force *F*_*c*_ (see Section 5.2) ensures the system maintains contact during the run duration. The protocol is straightforward, ensuring no training is necessary before performing these tests.

### 6.4 Protocol IV: Quasi-static displacement protocol

**FIGURE 11:**
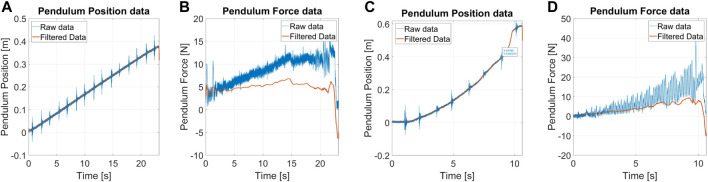
**(A)** angular position and **(B)** force estimation for the quasi-static displacement disturbance experiments, and **(C)** angular position and **(D)** force estimation for the quasi-static force disturbance experiments.

Tests are executed by providing a slow-varying ramp in the contact point position, with a slope of 1.5 *cm*/*s*. Experiments start after the pendulum contact algorithm and stop if the robot falls or exceeds the maximum allowable displacement. Figure 11 shows an example of the measurements for protocol IV. The force measurements also show the gravity rejection from raw to filtered data.

During the experiments, the robot did not show any unstable behavior, so it was not possible to define the QSR. Alter-Ego acts like an inverted pendulum mounted on a Segway. The system is regulated by an LQR controller, with high weights on the pitch dynamics and low authority on the position. This design choice is because we want the system not to fall, regardless of the position. This behavior reflects on the QSR, since the robot moves away from the desired position under the action of external frontal pushes, as the main task is to maintain stability.

### 6.5 Protocol V: Quasi-static force protocol

Tests are executed by providing a slow varying ramp of 1*N*/*s*. Experiments start right after the pendulum contact algorithm and stop if the contact is not preserved or the position exceeds the maximum allowable displacement.

Figure 11 shows examples of measurements for quasi-static force perturbation. As in the previous cases, we estimate the force through the torque sensor and correct the bias due to the pendulum weight. Oscillations of force are due to the friction created by sliding the pendulum tip on the robot covers. The robot did not show unstable behaviors, and, for this reason, it was not possible to define the QSR.

Regarding the discussion of the performance of Alter-Ego under quasi-static forces, the same considerations made on Section 6.4 hold.

## 7 Discussion

We verified the efficacy of the benchmarking method by quantifying the balancing performance of Alter-Ego. Results reported in Section 6 are promising in assessing systems resilience for many reasons. The experimental campaign exhibits a high degree of repeatability. Indeed, it was possible to perform a large number of experiments under the same conditions. Moreover, the standard deviation of each data set from the mean value shown in the PIs graphs is adequate to have a certain degree of statistical validity. As a result, we tested and thoroughly characterized the robot within a few days. The benchmark allows for easily switching testbed conditions and control techniques during the experimental campaign. Efforts done by operators during the protocol selection and execution are minimal since the control routine and parameters can be chosen by software at the beginning of each test. Protocol I routine is the lone exception, requiring the operator to change the pendulum’s inertia to obtain the appropriate conditions. To facilitate the procedure, we designed the system so that adding and removing masses is a simple process. The first calibration of the structure parameters (see Section 4.1) to match the dimensions of robots under testing requires a relatively low effort. H is adjusted by moving the structure using an electrical crane, making it a simple procedure. L and M are modified by changing the pendulum bar and adding masses. Moreover, tuning parameters is a preliminary procedure, and it is required to be performed once for a testing campaign (twice if experiments are performed both on the frontal and lateral planes) since the contact point is the same for all protocols. Lastly, the performance evaluation relies solely on the sensors integrated into the framework. Therefore, all the results are consistent, allowing us to compare different systems with a meaningful metric.

To improve the efficiency of tests, we define a method for selecting the optimal experimental conditions for the first protocol, allowing us to identify the experiments *a priori*. We used a mock model and Alter-Ego to validate the concept behind the experiment conditions. The reason behind this choice is that at least two systems must be used to ensure that this definition is reliable for most of the robots that will be tested. Figure 7B shows an example of conditions under which we should test the robot. The testbench automatically generates the required experiments that the operators must execute, indicating the necessary mass, pendulum length, and starting position in a matrix. By using normalization factors, the matrix is constructed by scaling a given set of initial values [*E*, *I*] to the robot size. Protocols II and III, on the other hand, already have a straightforward procedure. The starting values of the amplitude are determined by the robot dimensions, especially for force ranges.

The perturbations considered in this work do not describe the totality of disturbances that can be applied to a self-stabilizing robot but the ones that are the most common to the best of our knowledge. In future works, we are planning to define more testing protocols to account for more perturbations. Some examples can be found in sudden forces and displacements that last over time, occurring when a robot impacts heavy external objects, or pseudo-random force signals, possible while interacting with external operators.

## 8 Conclusion

In our work, we investigated the stability characterization of robotic systems subjected to external perturbations. We propose a benchmarking method for testing systems of different sizes with reliable and repeatable experiment conditions. To characterize robots performance quantitatively, we provide a set of protocols and performance indicators. The aim is to allow comparisons between different mechatronics solutions or the same system with distinct controllers. Finally, we propose a datasheet to summarize the balancing performance of robots resulting from experiments in our framework. We used the framework to characterize Alter-Ego, a two-wheeled robust humanoid robot, to evaluate the effectiveness of our benchmarking method. In this regard, we ran a campaign with 1120 tests. Quantitative evaluation of robot performance will promote the improvement of robots and push forward the standardization and regulation of these technologies.

## Data Availability

The datasets presented in this study can be found in online repositories. The names of the repository/repositories and accession number(s) can be found below: https://www.naturalmachinemotioninitiative.com/benchmark-robot-balancing Data can be found in download link on “Experimental data of AlterEgo characterization”.

## Footnotes

1
https://eurobench2020.eu/.

2 Agility Robotics, https://www.agilityrobotics.com.

3 Unitree robotics, https://www.unitree.com.

4 ANYbotics, https://www.anybotics.com.

5 Soft Bank Robotics, https://www.softbankrobotics.com.

6 Boston Dynamics, https://www.bostondynamics.com.
